# Microbiome-derived TOPOSCORE is associated with response to immune checkpoint inhibitor re-induction after chemotherapy in primary resistant metastatic melanoma

**DOI:** 10.1007/s00262-026-04618-2

**Published:** 2026-10-30

**Authors:** Sebastian Haferkamp, Bastian Schilling, Lea Baumgart, Anja Gesierich, Valerie Glutsch, Konstantin Drexler, Lisa Wein, Monika Morak, Michael Erdmann, Carola Berking, Gianmarco Piccinno, Lisa Derosa, Laurence Zitvogel, Ying Wang, Lucie Heinzerling

**Affiliations:** 1 Department of Dermatology, University Hospital Regensburg, Regensburg, Germany; 2 Department of Dermatology and Venereology, University Hospital Halle, Martin-Luther-University Halle-Wittenberg, Halle, Germany; 3 Bavarian Cancer Research Center (BZKF), Erlangen, Germany; 4 CCC Alliance WERA, Bavaria, Germany; 5 Department of Dermatology, Venereology and Allergology, Goethe University Frankfurt, University Hospital, Frankfurt Am Main, Germany; 6 Department of Dermatology and Allergy, LMU Medizin, LMU University Hospital, Ludwig-Maximilians-Universität München, Frauenlobstr. 9-11, 80337 Munich, Germany; 7 Department of Dermatology, Venereology and Allergology, Bavarian Cancer Research Center (BZKF), University Hospital Würzburg, Würzburg, Germany; 8 Department of Dermatology, Deutsches Zentrum Immuntherapie (DZI), CCC Erlangen-EMN, Friedrich-Alexander-Universität Erlangen-Nürnberg (FAU), Uniklinikum Erlangen, Erlangen, Germany; 9 Department of Cellular, Computational and Integrative Biology (CIBIO), University of Trento, Trento, Italy; 10 Clinicobiome, Gustave Roussy, Villejuif, France; 11 Université Paris-Saclay, Villejuif, France; 12 Équipe Labellisée – Ligue Nationale contre le Cancer, Institut National de La Santé Et de La Recherche Médicale (INSERM) U1015, Villejuif, France

**Keywords:** Melanoma, TOPOSCORE, Microbiome, Chemotherapy, Immune checkpoint inhibitors, Rechallenge

## Abstract

**Background:**

Primary resistance to immune checkpoint inhibitors (ICIs) remains a major challenge in metastatic melanoma. The prospective multicenter PROMIT trial showed that intermittent chemotherapy could restore sensitivity to ICI re-induction in a subset of patients. We investigated whether gut microbiome ecosystem states characterized by TOPOSCORE were associated with tumor response in this setting.

**Methods:**

In the phase II PROMIT trial (NCT04225390), 24 patients with longitudinal stool samples were included in this exploratory microbiome analysis. ICI-resistant patients received two cycles of dacarbazine followed by ICI re-induction. Stool samples underwent shotgun metagenomic sequencing. Microbiome ecosystem states were classified into SIG1- or SIG2-associated ecosystem states using the TOPOSCORE framework, which is based on species-interacting groups (SIGs) defined by ecological co-abundance patterns.

**Results:**

Baseline TOPOSCORE distributions and s-scores were not associated with clinical outcome. In the nine patients with complete BL-CTX-ICI sampling, six retained the same TOPOSCORE category throughout treatment and three changed category. Of the two patients with PR, one remained SIG2 throughout, whereas the other changed from SIG2+ to SIG1+ after chemotherapy and returned to SIG2+ during ICI re-induction. Analyses using all available samples at each phase likewise did not show significant differences in s-score among PR, SD, and PD groups.

**Conclusions:**

Baseline TOPOSCORE classification was insufficient to predict treatment outcome in this cohort. Longitudinal TOPOSCORE patterns were heterogeneous, and the small number of complete longitudinal profiles precludes conclusions that chemotherapy induced a favorable microbiome transition. These exploratory findings support further evaluation of microbiome ecosystem dynamics in larger prospective cohorts.

**Supplementary Information:**

The online version contains supplementary material available at https://doi.org/10.1007/s00262-026-04618-2.

## Introduction

Primary resistance to immune checkpoint inhibitors (ICIs) in *BRAF* wild-type patients with metastatic melanoma poses a major clinical challenge, as it is associated with poor prognosis. The underlying mechanisms are complex and heterogenous, including tumor-intrinsic alterations, immune cell dysfunction, unfavorable gut microbiota and an immunosuppressive tumor microenvironment. Understanding these interactions and identifying strategies capable of changing the microbiome and overcoming resistance by restoring responsiveness in this high-risk patient group represent a significant medical need [1, 2].

The gut microbiome has emerged as key regulator of systemic immune function and a determinant of response to cancer immunotherapy [3–6]. Accumulating evidence from preclinical and clinical studies suggests that the composition and ecological structure of the gut microbiota, such as increased gut microbial alpha-diversity and higher relative abundance of Faecalibacterium, Akkermansia and Bifidobacterium species, promote antitumor immunity and improve clinical outcomes during ICI therapy. In contrast, non-responders exhibit distinct gut microbial communities characterized by depletion of beneficial commensals and enrichment of potentially pro-inflammatory taxa, which are associated with impaired immune responses [4, 7]. However, despite extensive investigation in melanoma, no reproducible microbiome signature across independent cohorts has been established that reliably stratifies patients at an individual level [8–10]. High interindividual variability, geographic and technical heterogeneity, and the dynamic nature of the microbial ecosystems have hampered clinical translation of microbiome-based biomarkers [11]. In addition, comprehensive microbiome profiling remains methodologically complex, relying on advanced sequencing and bioinformatic approaches that limit feasibility in routine oncology practice. Consequently, there is a growing need for robust and user-friendly tools that enable clinically meaningful microbiome stratification and may support treatment stratification and microbiota-centered therapeutic strategies [12].

To address these limitations, Derosa et al. developed the TOPOSCORE, an ecosystem-based gut dysbiosis score derived from species-level co-abundance network analysis [12]. In this framework, microbial gene species cluster into species-interacting groups (SIGs) that reflect distinct ecological community states. Integration of these ecological signatures with Akkermansia species abundance enables calculation of an individualized TOPOSCORE, capturing cooperative microbial ecosystem structures rather than isolated bacterial taxa. Distinct TOPOSCORE states have been associated with differential outcomes under ICI therapy [12].

Whether TOPOSCORE retains clinical relevance in patients with metastatic melanoma exhibiting primary resistance to ICIs remains unknown. Cytotoxic chemotherapy may influence several determinants of antitumor immunity, including tumor antigenicity, antigen presentation, immunosuppressive immune-cell populations, and the intestinal microbiome. Preclinical and clinical studies indicate that chemotherapy can alter gut microbial composition and that microbiota-dependent immune responses may contribute to therapeutic effects [13–15]. However, whether such remodeling occurs after dacarbazine and contributes to renewed responsiveness to ICI remains unclear. Therefore, we investigated TOPOSCORE dynamics in longitudinal stool samples obtained during the prospective PROMIT trial and explored the relationship between microbial ecosystem states, temporal microbiome transitions, and clinical outcomes following chemotherapy and ICI re-induction.

## Methods

### Study design and patient population

Patients with primary ICI-resistant unresectable BRAF wildtype metastatic melanoma were enrolled in the prospective, multicenter phase II PROMIT trial (NCT04225390), as previously reported [16]. All patients gave written informed consent. Eligible patients had received prior ICI, either anti–PD-1/PD-L1 monotherapy or combined CTLA-4/PD-1 blockade, and were required to never have responded to the administered ICI regimen. Following confirmation of primary resistance defined as stable disease or disease progression, patients received two cycles of dacarbazine (DTIC, 850 mg/m^2^ intravenously), administered three weeks apart. Subsequently, patients were re-induced with the same ICI regimen to which they had previously exhibited progressive disease (PD).

### Stool sample collection and microbiome analysis

Stool samples were prospectively collected at predefined time points: at baseline prior to chemotherapy, after completion of dacarbazine treatment, and during ICI re-induction. Fecal samples were collected in DNA/RNA Shield Fecal Collection Tubes according to the manufacturer’s instructions and stored at − 20 °C until processing. For metagenomic analysis, total DNA was extracted from stool samples and subjected to shotgun metagenomic sequencing at Prebiomics. Taxonomic profiling was performed using MetaPhlAn 4 with the corresponding ChocoPhlAn reference database, followed by downstream microbiome analyses according to the established TOPOSCORE pipeline [12]. Using an ecosystem-based approach, species-level co-abundance networks were constructed from metagenomic sequencing data, enabling classification of microbial communities into SIGs with opposing clinical relevance. Integration of these ecological signatures, including the quantification of *Akkermansia* species, allowed for calculation of an individualized topological score (TOPOSCORE), reflecting the dominant microbial ecosystem state. Previous studies reported associations between SIG1/SIG2 states and clinical outcomes [12]. Sample-level TOPOSCORE data for all 51 analyzed stool samples are provided in Supplementary Data 1.

Tumor response was assessed according to RECIST version 1.1 (complete response [CR], partial response [PR], stable disease [SD], or progressive disease [PD]), as previously reported for the PROMIT trial [16]. For response-group analyses, responders were defined as patients with CR or PR. No CR occurred in the microbiome cohort; therefore, the responder group consisted of patients with PR. Patients with SD or PD were considered non-responders. Analyses at individual baseline (BL), at chemotherapy/immunotherapy transition (CTX), and ICI treatment (ICI) phases used all available samples at that phase. Complete-case longitudinal trajectory analyses were restricted to patients with samples available at all three phases.

### Exploratory fecal metabolomic profiling

Exploratory fecal metabolomic profiling was performed in available PROMIT stool samples collected across longitudinal sampling time points from screening to week 13. Metabolites were analyzed by the Gustave Roussy metabolomics platform using complementary targeted and semi-targeted mass spectrometry approaches, including UHPLC–QQQ, GC–QQQ, and UHPLC–Orbitrap platforms. After quality control filtering, 153 metabolites were retained for exploratory analysis. Metabolite abundances were corrected for analytical bias, log2-transformed, and mean-centered. For samples available from the same patients at both compared time points, paired comparisons were performed using the Wilcoxon signed-rank test. Given the exploratory nature of the metabolomic analysis and the limited number of paired samples, no adjustment for multiple testing was applied. Accordingly, all metabolomic findings were considered nominal, exploratory, and hypothesis-generating.

### Statistical analysis

Associations between TOPOSCORE s-scores and clinical response were evaluated using the Kruskal–Wallis test, and categorical TOPOSCORE distributions were evaluated using Fisher's exact test. In addition to the prespecified baseline analysis, exploratory cross-sectional analyses were performed using all available samples at CTX and ICI phases. Complete-case longitudinal TOPOSCORE trajectories were visualized to assess within-patient temporal changes. Due to the limited sample size, longitudinal analyses were considered exploratory and descriptive. Statistical analyses and graphical visualizations were performed using GraphPad Prism version 10.1.1 and R version 4.5.1. A two-sided *p* value < 0.05 was considered statistically significant.

## Results

### Longitudinal stool sampling of the PROMIT cohort

**Fig. 1:**
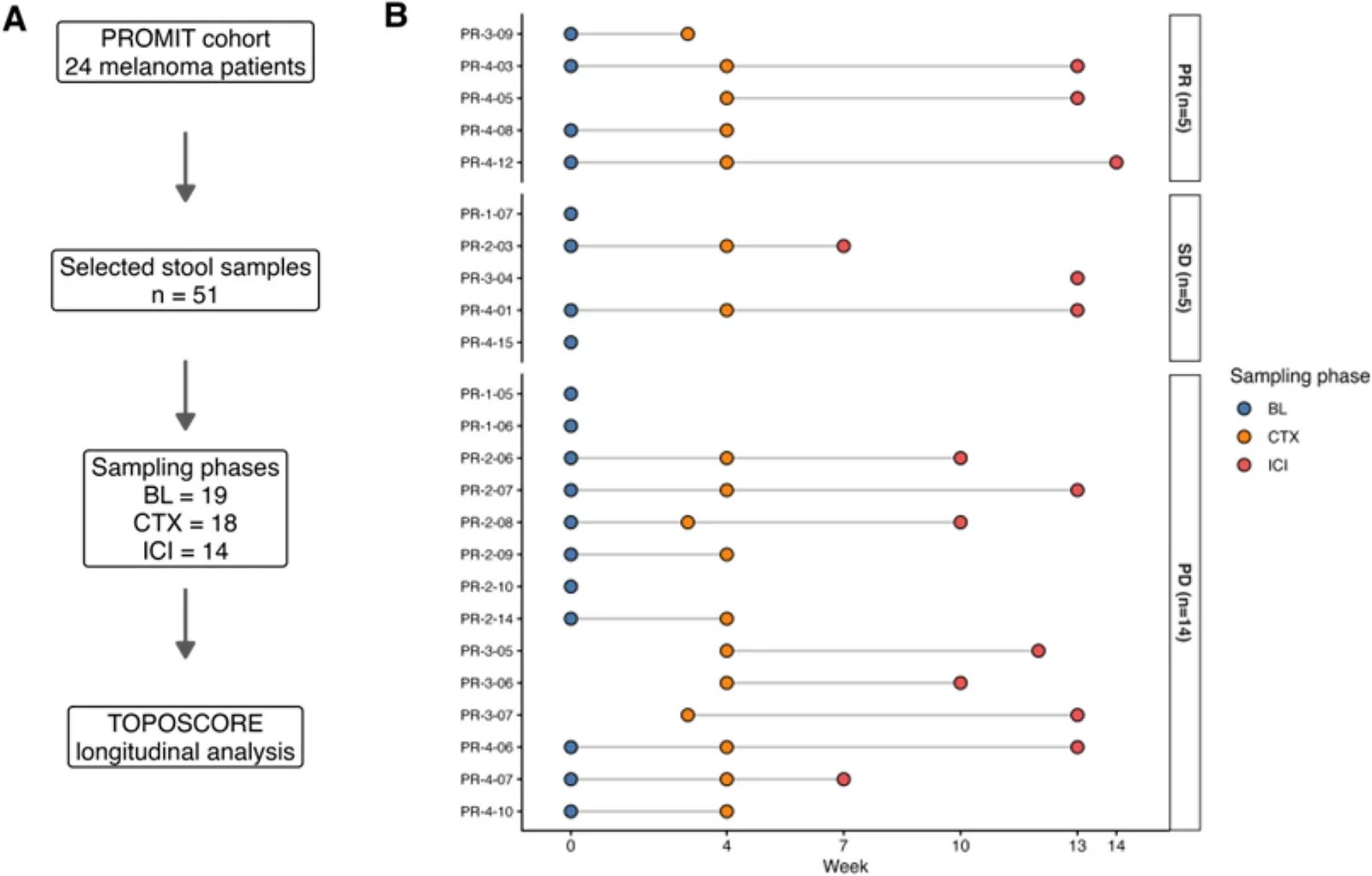
Longitudinal stool sampling across the PROMIT melanoma cohort. Each row represents an individual patient. Colored circles indicate stool collection time points and are connected by grey lines to illustrate longitudinal sampling. Patients are grouped according to best clinical response: partial response (PR, n = 5), stable disease (SD, n = 5), and progressive disease (PD, n = 14). Stool samples were obtained at baseline (BL), during chemotherapy/immunotherapy transition (CTX), and during immune checkpoint inhibitor treatment (ICI), resulting in a total of 51 samples from 24 patients

A total of 24 melanoma patients treated with ICIs were included in this study. Fifty-one stool samples were collected longitudinally across baseline (BL, n = 19), chemotherapy/immunotherapy transition (CTX, n = 18), and on ICI treatment time points (ICI, n = 14). In this cohort, clinical responses included 5 patients with partial response (PR), 5 with stable disease (SD), and 14 with progressive disease (PD) (Fig. 1A). Longitudinal sampling enabled assessment of temporal changes in gut microbiome TOPOSCORE signatures throughout treatment (Fig. 1B).

### Baseline TOPOSCORE was not associated with clinical response

**Fig. 2:**
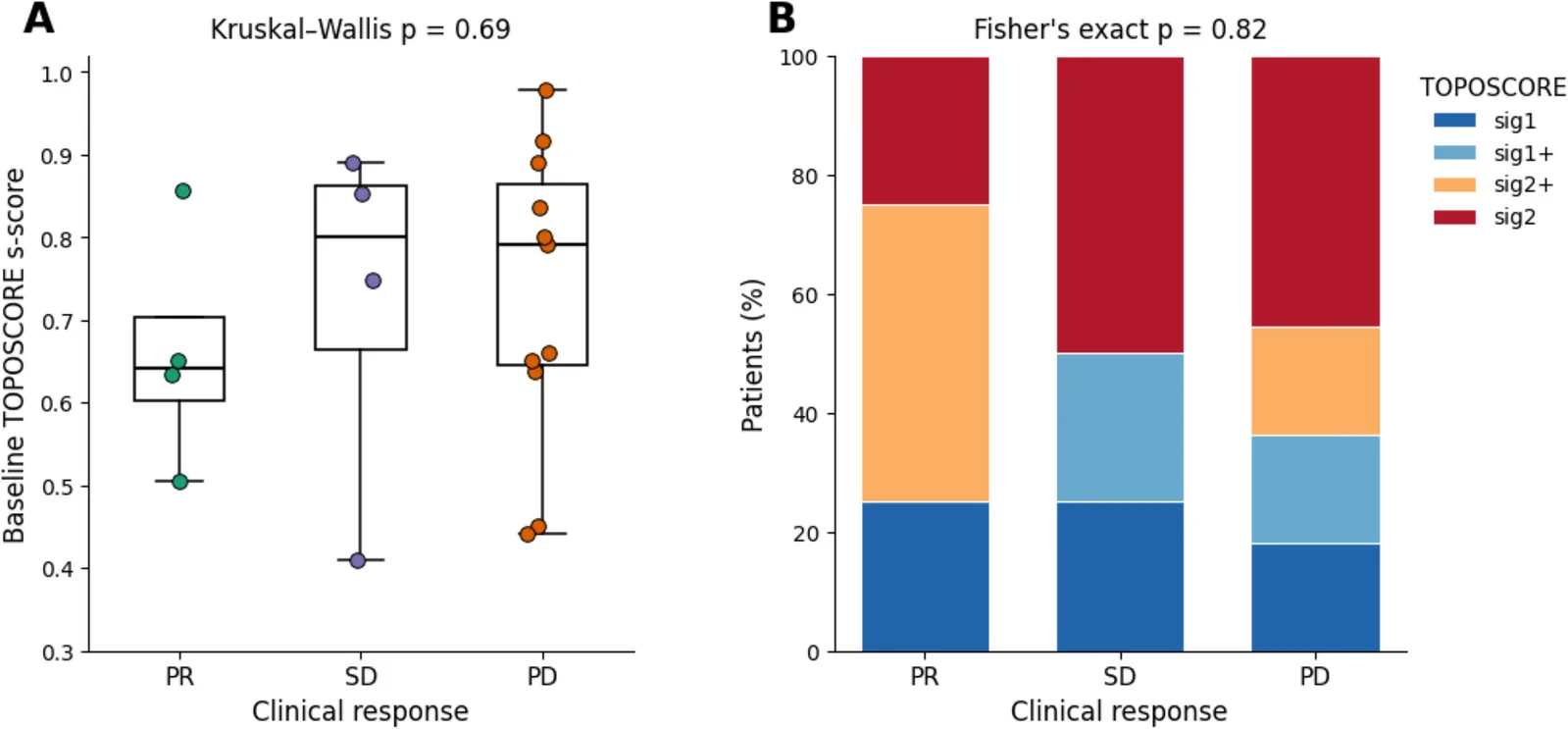
Baseline TOPOSCORE is not associated with clinical response in melanoma patients receiving immune checkpoint inhibitors. **A** Distribution of baseline TOPOSCORE s-scores according to best clinical response. Individual patients are shown as dots and boxplots indicate the median and interquartile range. No significant difference was observed among patients with partial response (PR), stable disease (SD), or progressive disease (PD) (Kruskal–Wallis test, *p* = 0.69). **B** Distribution of baseline TOPOSCORE categories (SIG1, SIG1+, SIG2+, and SIG2) across clinical response groups. Stacked bars represent the proportion of patients within each response category. No association between baseline microbial topology and clinical response was detected (Fisher’s exact test, *p* = 0.82)

To determine whether baseline gut microbial topology was associated with treatment outcome, TOPOSCORE was evaluated in pretreatment stool samples. Baseline s-scores did not differ significantly among patients achieving PR, SD, or PD (Kruskal–Wallis test, *p* = 0.69; Fig. 2A). Likewise, the distribution of TOPOSCORE categories (SIG1, SIG1+, SIG2+, and SIG2) was comparable across response groups, with no significant enrichment of a specific microbial topology among responders or non-responders (Fisher’s exact test, *p* = 0.82; Fig. 2B). Although four of five responders (80%) exhibited a SIG2-associated microbiome state at baseline, similar microbial states were also observed among non-responders. Taken together, these findings indicate that baseline TOPOSCORE alone was insufficient to predict clinical response to ICI re-induction in the PROMIT study.

### Longitudinal TOPOSCORE dynamics during chemotherapy and ICI re-induction

**Fig. 3:**
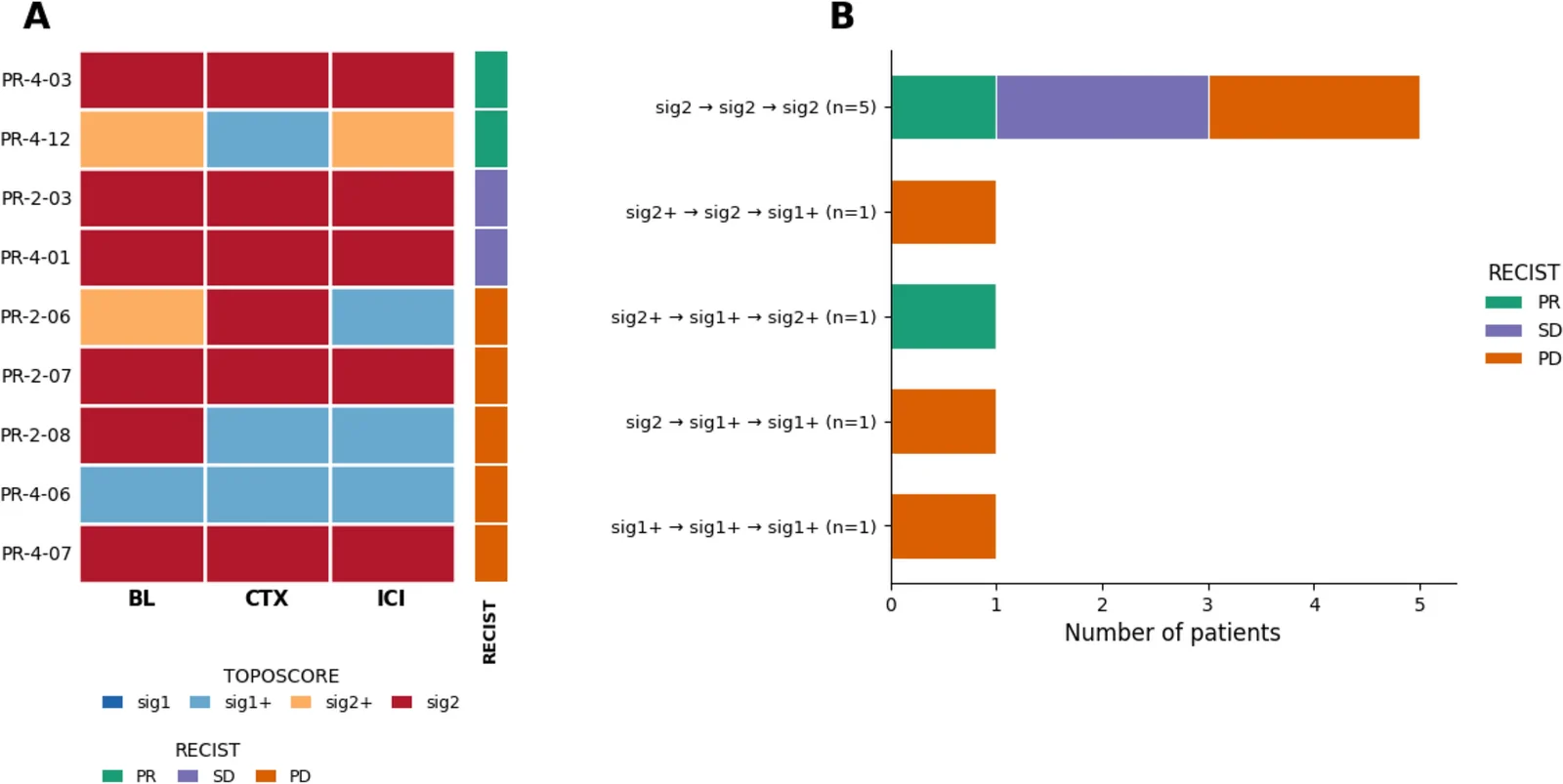
Longitudinal TOPOSCORE dynamics during chemotherapy and immune checkpoint inhibitor re-induction. **A** TOPOSCORE classifications at baseline (BL), after chemotherapy (CTX), and during ICI re-induction (ICI) in the nine patients with complete longitudinal sampling; RECIST response is indicated for each patient. **B** Frequency of observed TOPOSCORE trajectory patterns, stratified by RECIST response (PR, SD, PD). Six of nine patients retained the same category across all three phases, while three changed category

Longitudinal trajectory analysis was restricted to nine patients with complete stool sampling (patient-level clinical characteristics and ICI re-induction regimens are summarized in Supplementary Table 1) at baseline (BL), after chemotherapy (CTX), and during ICI re-induction (ICI). Six of nine patients (66.7%) retained the same TOPOSCORE category across all three phases, whereas three (33.3%) changed category (Fig. 3A-B). The three non-stable trajectories were SIG2+ -> SIG2 -> SIG1+ (PD), SIG2 -> SIG1+ -> SIG1+ (PD), and SIG2+ -> SIG1+ -> SIG2+ (PR). Thus, one PR patient remained SIG2 throughout treatment and the second PR patient showed a transient shift to SIG1+ after chemotherapy before returning to SIG2+ during ICI re-induction. These observations do not support a uniform chemotherapy-induced shift toward SIG2 and should be interpreted descriptively given the small number of complete longitudinal profiles. To address potential loss of information from complete-case restriction, we additionally examined all available samples at each phase. TOPOSCORE s-scores did not differ significantly among PR, SD, and PD groups at BL (n = 19, Kruskal–Wallis *p* = 0.69), CTX (n = 18, *p* = 0.29), or ICI (n = 14, *p* = 0.077). These phase-specific analyses are cross-sectional because the available patients differ between phases and therefore should not be interpreted as paired treatment effects. Clinical characteristics of the nine patients with complete longitudinal profiling, including the individual ICI re-induction regimen, are provided in Supplementary Table 1.

### Distribution of SIG-associated bacterial species across clinical response groups

**Fig. 4:**
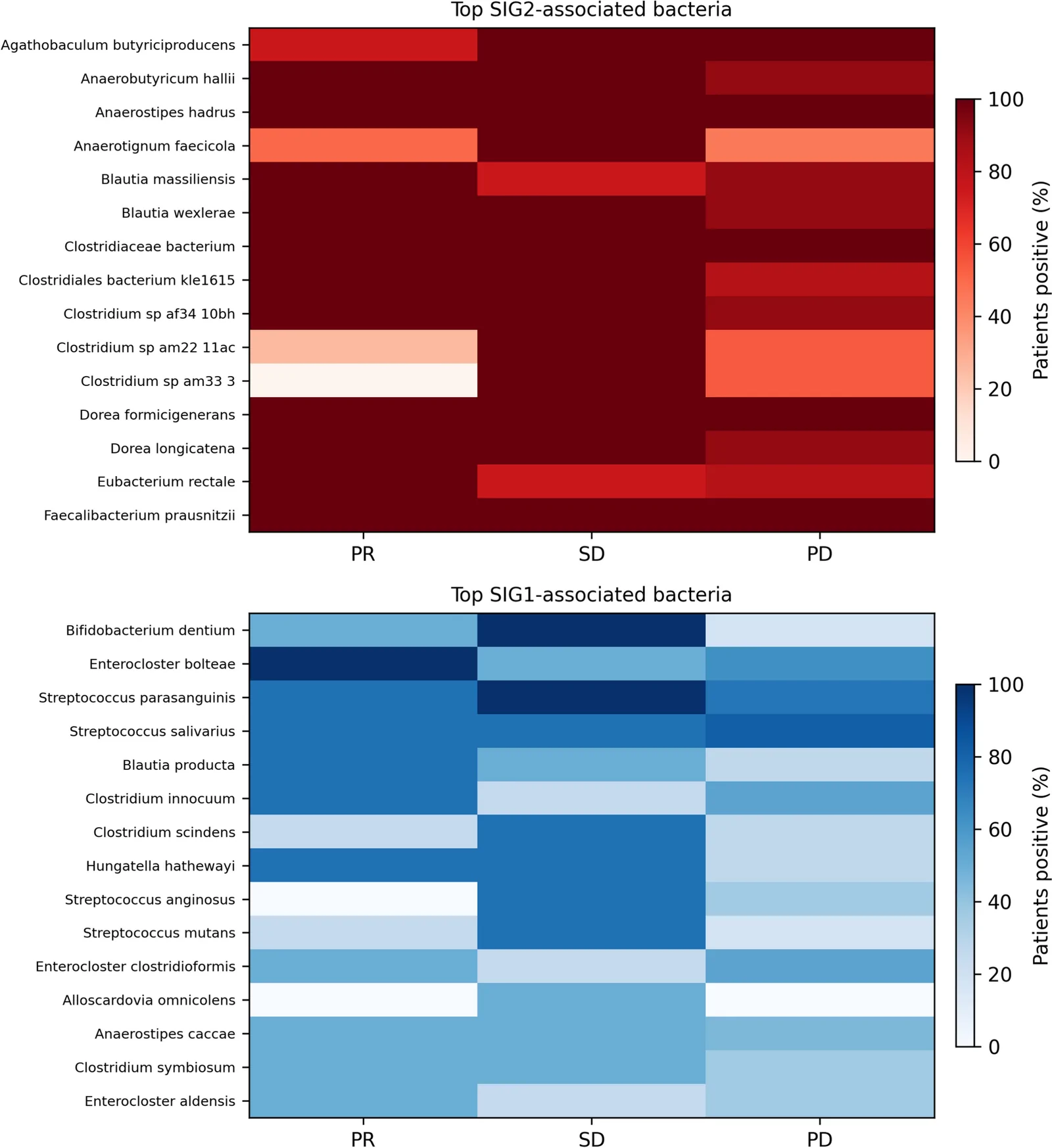
Patient-level prevalence of bacterial species included in the TOPOSCORE signatures. Top: prevalence of frequently detected SIG2-associated bacterial species among patients with partial response (PR), stable disease (SD), and progressive disease (PD). Bottom: prevalence of frequently detected SIG1-associated bacterial species. Values represent the percentage of patients within each response group carrying the indicated bacterial species. Separate 0–100% color scales are shown for both the red (SIG2) and blue (SIG1) panels

To descriptively characterize taxa contributing to the predefined TOPOSCORE signatures, we examined the prevalence of SIG1- and SIG2-associated bacterial species across clinical response groups. Frequently detected SIG2-associated taxa included Faecalibacterium prausnitzii, Roseburia intestinalis, Roseburia hominis, Eubacterium rectale, Anaerostipes hadrus, and Agathobaculum butyriciproducens, whereas frequently detected SIG1-associated taxa included Streptococcus salivarius, Streptococcus parasanguinis, Enterocloster bolteae, Clostridium innocuum, and Veillonella parvula (Fig. 4). These prevalence summaries are descriptive and should not be interpreted as differential-abundance testing between response groups.

### Exploratory fecal metabolomic profiling during PROMIT treatment

To complement the TOPOSCORE-based microbiome analysis, exploratory fecal metabolomic profiling was performed in available longitudinal PROMIT stool samples. After quality control, 153 metabolites were retained for analysis across seven sampling time points from screening to week 13. Overall, the fecal metabolomic landscape appeared largely stable over time. Exploratory paired analyses suggested nominal temporal changes in selected metabolites, particularly involving oxidized lipid metabolites, fatty acid-related metabolites, microbial fermentation-associated metabolites, and polyamine-related metabolites. These included 9-HOTrE, 9(S)-HODE/13(S)-HODE, linolenic acid, eicosenoic acid, glycerol, isobutyric acid, isovaleric acid, sebacic acid, gamma-glutamylthreonine, dopamine, and N1-acetylspermidine (Supplementary Fig. 1). No adjustment for multiple testing was performed; accordingly, the observed P values are nominal and these findings should be interpreted as exploratory and hypothesis-generating.

## Discussion

This exploratory study investigated microbiome-derived ecological states, as captured by the TOPOSCORE, during chemotherapy and subsequent ICI re-induction in patients with primary ICI resistance.

In this cohort of patients with primary resistance to ICIs, baseline TOPOSCORE alone was not associated with clinical response. Among the nine patients with complete longitudinal sampling, six retained the same TOPOSCORE category and three changed category. Importantly, the two evaluable PR patients followed different trajectories: one remained SIG2 throughout, whereas the other transiently changed from SIG2+ to SIG1+ after chemotherapy before returning to SIG2+ during ICI re-induction. Accordingly, these data should not be interpreted as evidence that dacarbazine consistently converts the microbiome toward a favorable SIG2-associated state. Rather, they show heterogeneous ecosystem trajectories that warrant validation in larger cohorts.

The observation that several responders already exhibited SIG2-associated states at baseline further argues against attributing treatment response to chemotherapy-induced microbiome conversion. Nevertheless, there is a biological rationale for investigating chemotherapy as a potential modifier of the host-microbiome-immune axis. Preclinical studies have shown that the efficacy and immunomodulatory effects of cytotoxic therapy can depend on commensal bacteria and microbiota-mediated regulation of the tumor microenvironment [14, 15]. In patients with non-haematological malignancies, longitudinal sampling has demonstrated transient chemotherapy-associated changes in gut microbial richness, diversity, and composition [13]. These findings support the concept that cytotoxic therapy can perturb intestinal microbial ecosystems, but they cannot be directly extrapolated to dacarbazine or to restoration of ICI sensitivity. Consistent with this uncertainty, our longitudinal TOPOSCORE data did not demonstrate a uniform dacarbazine-induced transition toward a favorable microbial ecosystem state. The present dataset therefore cannot establish a causal microbiome-mediated mechanism of resistance reversal, and these findings should be regarded as hypothesis-generating.

Notably, while numerous studies have linked individual bacterial taxa to ICI efficacy, reproducible and clinically applicable microbiome signatures for melanoma patients have remained elusive [17]. The TOPOSCORE represents a conceptually distinct approach by summarizing complex microbial interactions into an ecosystem-based score derived from species co-abundance networks. By integrating cooperative microbial communities rather than isolated taxa, this scoring system addresses key limitations of prior microbiome biomarker efforts, including lack of robustness and poor inter-cohort reproducibility [12]. Our data suggest that such an ecosystem-level classifier may retain clinical relevance even in a refractory disease setting and after cytotoxic therapy.

Several limitations warrant careful consideration. The cohort analyzed here is small, and only nine patients had complete BL-CTX-ICI stool sampling. Complete three-phase sampling was available for 2/5 PR, 2/5 SD, and 5/14 PD patients, indicating that missing samples occurred across all response groups. Missingness may not have been random and could have been associated with patient characteristics, treatment course, or clinical outcomes. In clinical practice, patients may be unable to provide stool samples at predefined visits due to clinical deterioration, disease progression, treatment discontinuation, or other factors affecting sample collection. However, the reasons for individual missing stool collections were not systematically documented in this cohort. Therefore, informative missingness and selection bias in the complete-case longitudinal analysis cannot be excluded. In addition, this Brief Report focuses on the predefined TOPOSCORE/SIG framework. The derived analysis dataset available for this study did not contain the full species-abundance and functional-profile matrices required for standard alpha- and beta-diversity, differential-abundance, or KEGG/MetaCyc/CAZy analyses. These analyses would require reprocessing of the underlying metagenomic data and should be incorporated into future validation studies. The exploratory metabolomic analysis was not sufficiently powered for integrative latent-variable modeling with the metagenome. These constraints limit mechanistic inference and preclude attributing observed TOPOSCORE changes specifically to dacarbazine.

Despite these limitations, this study provides an exploratory evaluation of TOPOSCORE-defined microbiome states in a prospective resistance-breaking treatment setting. The findings support further investigation of ecosystem-level microbiome measures together with conventional ecological and functional metagenomic analyses in larger, independently validated cohorts.

The present data therefore support TOPOSCORE as a hypothesis-generating framework rather than a stand-alone predictive biomarker or evidence of chemotherapy-induced microbiome remodeling.

In conclusion, baseline TOPOSCORE was not associated with response to ICI re-induction in this cohort, and longitudinal trajectories were heterogeneous. Larger studies with complete longitudinal sampling and complementary taxonomic, ecological, functional, and metabolomic analyses are required to determine whether microbiome dynamics contribute to restored ICI sensitivity after chemotherapy.

## Supplementary Information

Below is the link to the electronic supplementary material.

Supplementary file1 (XLSX 4 KB)

Supplementary file2 (CSV 57 KB)

Supplementary file3 (DOCX 192 KB)

## Data Availability

The processed sample-level dataset underlying the TOPOSCORE analyses is provided as Supplementary Data 1. Raw metagenomic sequencing data and associated metadata are not deposited with this revision and remain subject to applicable consent, data-governance, and institutional requirements. Access to additional data may be requested from the corresponding author, subject to these requirements.

## Abbreviations

CR: Complete response
CTLA-4: Cytotoxic T-lymphocyte-associated protein 4
CTX: Chemotherapy
DTIC: Dacarbazine
ICI: Immune checkpoint inhibitor
PD: Progressive disease
PD-1: Programmed cell death protein 1
PR: Partial response
RECIST: Response evaluation criteria in solid tumors
SD: Stable disease
SIG: Species-interacting group
SIG1: Species-interacting group 1
SIG2: Species-interacting group 2
TOPOSCORE: Topological dysbiosis score
