# Supplementary material for: Microbiome-derived TOPOSCORE is associated with response to immune checkpoint inhibitor re-induction after chemotherapy in primary resistant metastatic melanoma
Source: Cancer Immunol Immunother. 2026 Oct 30;75(11):265. doi: 10.1007/s00262-026-04618-2 (PMC13723481; doi:10.1007/s00262-026-04618-2)
Supplement: Supplementary file 1 [file 262_2026_4618_MOESM3_ESM.docx]

**Microbiome-derived TOPOSCORE is associated with response to immune checkpoint inhibitor re-induction after chemotherapy in primary resistant metastatic melanoma**

Suppl. Fig. 1

**Suppl. Fig 1. Longitudinal fecal metabolomic remodeling during PROMIT treatment.** (A) Heatmap of selected fecal metabolites exhibiting temporal alterations across seven longitudinal sampling time points (Screening, Week 1, Week 3, Week 4, Week 7, Week 10, and Week 13). Values represent row-scaled, QC-corrected, log2-transformed, mean-centered metabolite abundances (Z-scores). (B) Boxplots of four representative metabolites showing longitudinal changes during PROMIT treatment. Each dot represents one individual sample. Boxes indicate the interquartile range (IQR), center lines represent the median, and whiskers extend to 1.5 × IQR. Nominal P values are shown for representative paired comparisons and were calculated using Wilcoxon signed-rank tests including only patients with samples available at both compared time points. The number of paired samples (n) is indicated for each comparison. No adjustment for multiple testing was applied; therefore, all metabolomic findings are exploratory and hypothesis-generating.
